# Supplementary material for: The Structure of Maltooctaose-Bound Escherichia coli Branching Enzyme Suggests a Mechanism for Donor Chain Specificity
Source: Molecules. 2023 May 27;28(11):4377. doi: 10.3390/molecules28114377 (PMC10254366; doi:10.3390/molecules28114377)
Supplement: Supplementary file 1 [file molecules-28-04377-s001.zip › molecules-2366539-supplementary.pdf]

Supporting Information

The Structure of Malto-Octaose bound *Eschericia coli* Branching Enzyme Suggests a Mechanism for Donor Chain Specificity

Table of contents

A. Figure S1 3-4
B. Figure S2 5
C. Figure S3 6
D. Figure S4 7
E. Crystallographic Table 8

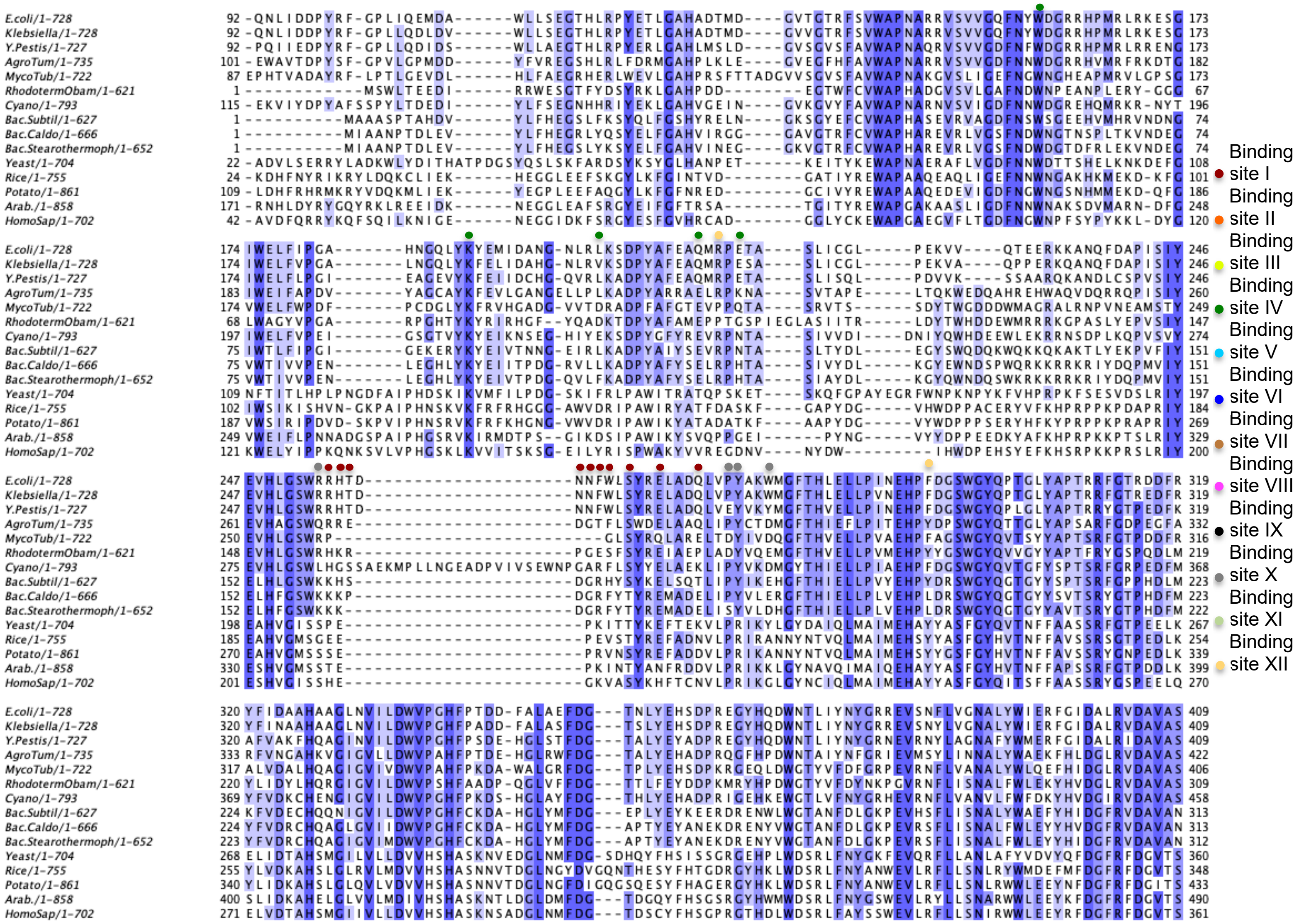
Binding site I
Binding site II
Binding site III
Binding site IV
Binding site V
Binding site VI
Binding site VII
Binding site VIII
Binding site IX
Binding site X
Binding site XI
Binding site XII

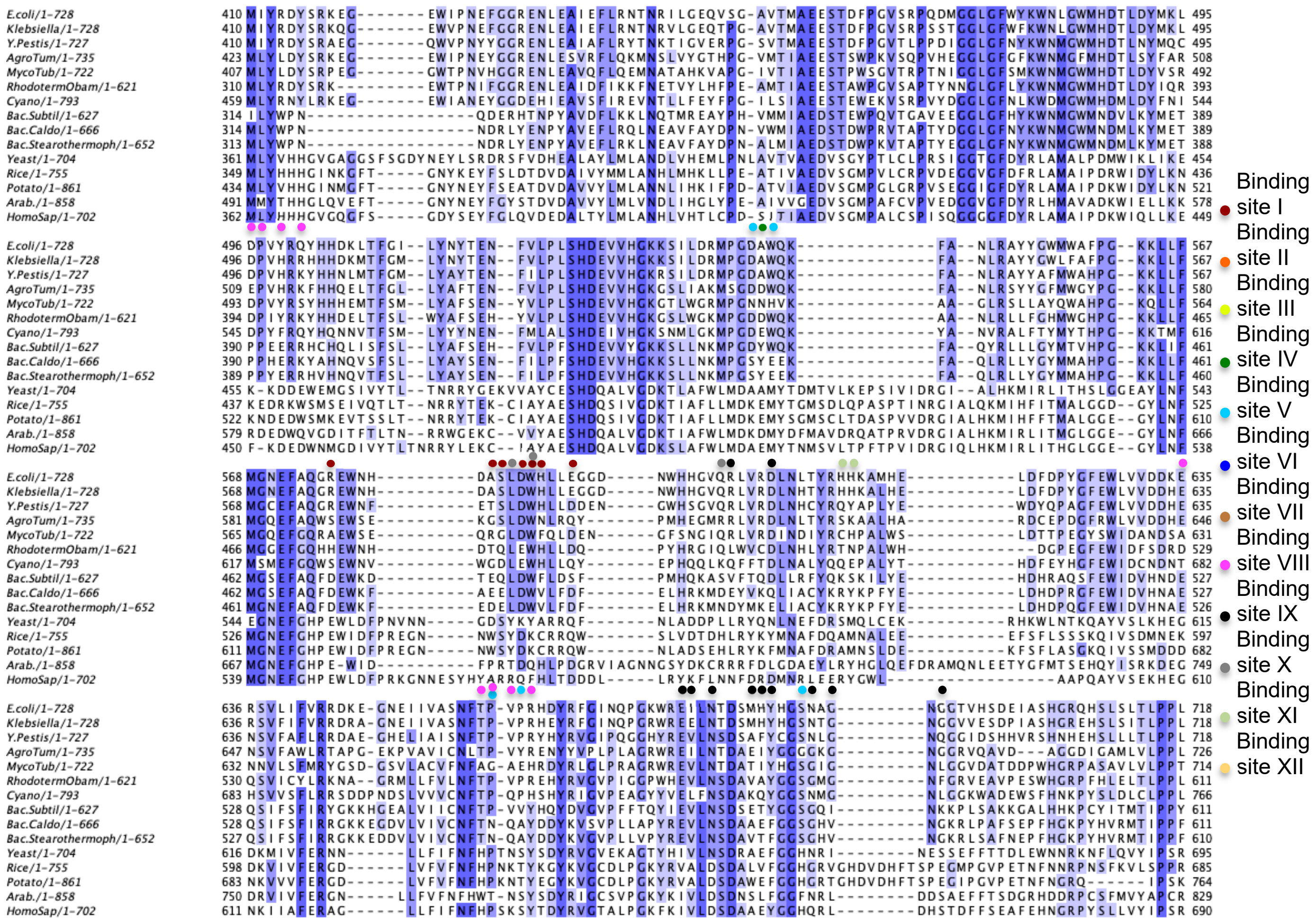

Figure S1. Sequence alignment of Branching Enzymes from various organisms. Identical residues are shown in blue. Amino acids in each binding site are shown.

(a) (b)

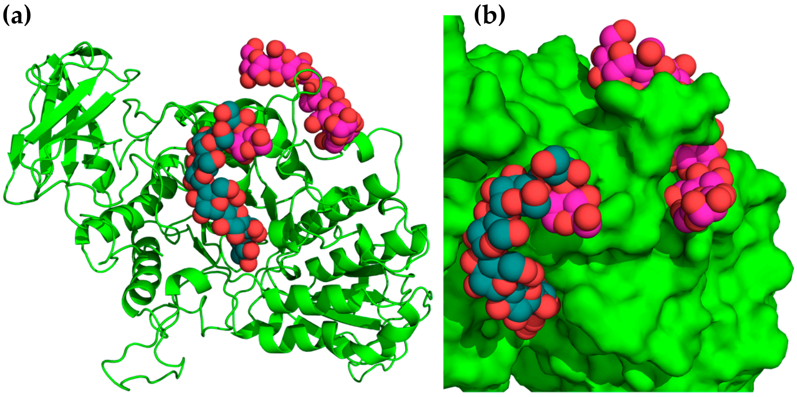

Figure S2. Site XII is close to the path of the donor chain. **(a)** The glucans bound in site I and XII (spheres, C, pink) of M8-bound EcBE (green ribbon) are shown with the M7 donor chain (spheres, C, blue-green) from the *Cyanothece* BE structure modeled into the structure. **(b)** EcBE is represented as a surface.

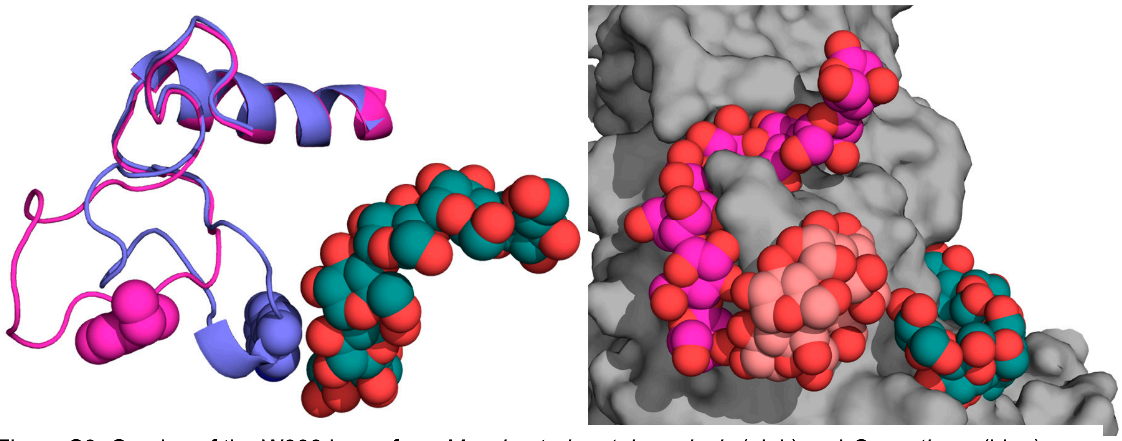

Figure S3. Overlay of the W399 loops from *Mycobacterium tuberculosis* (pink) and *Cyanothece* (blue) BE's. The active-site bound M7 donor chain from *Cyanothece* is shown as spheres, with C atoms colored blue-green. Right, M8-bound EcBE (grey surface) showing the site one bound glucan (Pink C atoms), with the cyclodextrin bound in site from the alpha-CD-bound EcBE structure (light red C atoms) and the *Cyanothece* M7 donor chain modeled onto the structure.

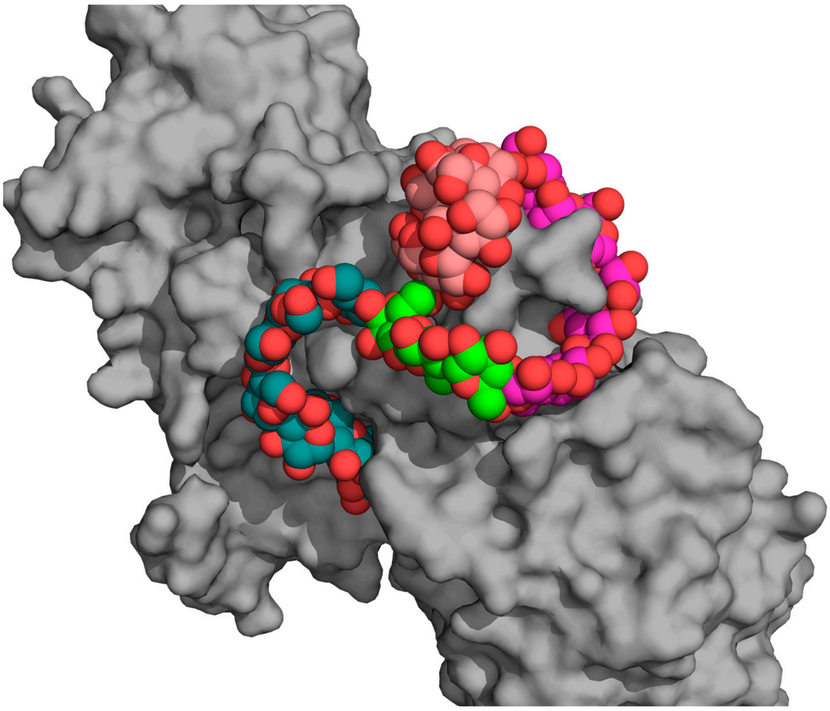

Figure S4. M8-bound EcBE structure showing site I-bound M8 (spheres, C, pink) with alpha-CD modeled into site VII (based on the alpha-CD bound EcBE structure, spheres, C, light red), active site bound M7 from *Cyanothece* BE modeled into the active site (spheres, C, blue-green) representing the donor strand and three malto-oligosaccharides connecting M8 and the modeled donor strand showing how site I could represent the binding surface of the longer donor strand characteristic of *E. coli* BE.

Table SI. Data collection and refinement statistics M8-bound EcBE.

**Wavelength (Å)**	0.978
**Resolution Range (Å)**	38.85 – 3.00
**Space group**	P3221
**a (Å)**	146.489
**b (Å)**	146.489
**c (Å)**	294.92
**a (°)**	90
**b (°)**	90
**γ (°)**	120
**Molecules per Asymmetric Unit**	4
**Total reflection**	1065049
**Unique Reflection**	71397
**Multiplicity**	2.1
**Completeness (%)**	96.33
**Average I/s**	22.9
R_{merge} **(%)**	0.14
R_{work} **(%)**	0.2139
R_{free} **(%)**	0.3152
**Reflections used in refinement**	71269
**Reflections used for** R_{free}	3615
**RMSD from ideal values**	
**Bond Angle**	1.36
**Bond Length (Å)**	0.012
**Average B factor**	69.29
**Number of water molecules**	473
**PDB ID**	**8SDB**
